# Musculoskeletal Disorders of the Upper Extremities Due to Extensive Usage of Hand Held Devices

**DOI:** 10.1186/s40557-014-0022-3

**Published:** 2014-08-06

**Authors:** Deepak Sharan, Mathankumar Mohandoss, Rameshkumar Ranganathan, Jeena Jose

**Affiliations:** 1 Department of Orthopaedic Surgery and Rehabilitation, RECOUP Neuromusculoskeletal Rehabilitation Centre, # 312, 10th Block, Further Extension of Anjanapura Layout, Bangalore 560062, Karnataka, India; 2 Department of Physiotherapy, RECOUP Neuromusculoskeletal Rehabilitation Centre, # 312, 10th Block, Further Extension of Anjanapura Layout, Bangalore 560062, Karnataka, India

**Keywords:** Hand held devices, Mobile phones, Myofascial pain syndrome, Tendinosis, Blackberry thumb, Text message injury

## Abstract

**Objective:**

The use of hand held devices (HHD) such as mobile phones, game controls, tablets, portable media players and personal digital assistants have increased dramatically in past decade. While sending a text message or using the controls of the HHD the users need to use their thumb and other palm muscles extensively. The objective of this study was to describe the risk factors and clinical features of the musculoskeletal disorders (MSDs) arising due to usage of hand held devices and to evaluate the effectiveness of a sequenced rehabilitation protocol.

**Methods:**

A retrospective report analysis of 70 subjects, who were diagnosed to have a MSD affecting the upper extremities, was conducted. Medical charts from a tertiary level rehabilitation centre from 2005–2013 were analysed. All the subjects reported pain in their upper extremities following extensive usage of HHD and were examined and diagnosed to have a MSD by an orthopaedic and rehabilitation physician. After the assessment and diagnosis, all the patients underwent rehabilitation using a sequenced protocol.

**Results:**

All the subjects reported pain in the thumb and forearm with associated burning, numbness and tingling around the thenar aspect of the hand, and stiffness of wrist and hand. 43 subjects had symptoms on the right side; 9 on left and 18 had bilateral symptoms. Correlation was found between hand dominance and MSD. 33 subjects complained of onset of symptoms following extensive text messaging. All the subjects were diagnosed to have tendinosis of Extensor Pollicis Longus and Myofascial Pain Syndrome affecting the 1st interossei, thenar group of muscles and Extensor Digitorum Communis. 23 of the subjects were senior executives, among these 7 were CEO’s of major multinational companies in India. All the subjects recovered completely following the rehabilitation.

**Conclusions:**

The study concluded that mobile phones and gadgets that promoted the predominant usage of thumb or only one finger while texting or using the controls were associated with a higher prevalence of MSDs. Treatment using a sequenced rehabilitation protocol was found to be effective.

## Introduction

Hand-held devices (HHD) are those devices which are used for communication and entertainment purposes such as media, internet access and gaming [1]. Also the multiple usability options available in the mobile phones encourage the users to engage a substantial period of his time in his HHD. The use of HHD is on the rise [2]. Mobile phone users are able to communicate other than by voice by a wide range of text button usage by means of SMS (short message service), whatsapp, viber, line, BBM (blackberry messenger) and social networking applications like facebook, twitter and skype. Texting is the most widely used mobile data service, with 74% of all mobile phone users worldwide being active users of it [3]. According to BBC reports, almost 19 billion messages were sent per day using chat apps and 17.6 billion SMS messages in 2012 [4]. Literature reports an adverse impact on the physical and psychological health of the users of HHD [5]. The incidence of musculoskeletal disorders (MSD) of hand, wrist, forearm, arm and neck has been increasing all over the world due to prolonged, forceful, low amplitude, repetitive use of hand held devices [6]. Sustained and gripping and repetitive movements with the thumb and fingers have all been identified as risk factors which may lead to disorders of the thumb and thumb musculature in the forearm. The range of movements of the thumb varies according to the size of the mobile and orientation of the keys [7]. Studies have shown a relation between mobile design and anthropometry of the user in causing discomfort and fatigue in hand, elbow and shoulder while using the HHD [8]. Additional factors include small spacing in the keypad, increased static loading, end-range motion of the thumb during texting and a difference in the muscle activity between individuals with and without musculoskeletal symptoms [9]. Phrases have been coined to describe MSD due to use of HHD such as ‘SMS thumb’, ‘iPod finger’, ‘blackberry thumb’, ‘wii injury’ and ‘nintenditis’; however, little evidence exists to support this association [10],[11]. Few studies in the recent years have reported about this growing problem that has a large impact globally. Hence, this study was conducted to describe the risk factors and clinical features of the MSD’s due to usage of HHD and to evaluate the effectiveness of a sequenced rehabilitation programme.

## Material & methods

A retrospective report analysis in which reports of 70 subjects between the ages of 5 to 56 years, who were diagnosed to have a MSD affecting the upper extremities were analysed. Reports of a tertiary level rehabilitation centre in Bangalore, India between the years of 2005 to 2013 were reviewed for the subjects reporting musculoskeletal pain in their upper extremities following extensive usage of HHD like mobile phones, game controls and tablets. The collected reports were analysed. The subjects with symptoms were all clinically examined and diagnosed by a single orthopaedic and rehabilitation physician. A subjective questionnaire was used to collect details about hand dominance, type of HHD used, total hours of usage per day and type of activity predominantly done using the HHD. The inclusion criteria was sending a minimum of 25 text messages or emails per day, browsing the Internet or playing games for more than 1 hour per day using the HHD, which was followed by the onset of symptoms. After the diagnosis and assessment, all the patients underwent rehabilitation for 2 to 4 weeks using a sequenced protocol. The sequenced protocol included a four phase model based on the pain level. Phase I included soft tissues mobilisation techniques (trigger point release, myofascial release, positional release technique, muscle energy technique); gentle grade 1 and 2 mobilisation of the upper extremity for pain; range of motion exercises for the elbow, wrist and finger joints and modalities such as ultrasound, low level laser therapy, contrast bath and taping. Phase II included gentle active and passive stretching of the muscles of upper extremity especially hand; hand exercises inside a water tub (Hydrotherapy); EMG Biofeedback for retraining the muscle during usage of HHD and ergonomic modification. Phase III included strengthening of the upper extremity muscles especially the hand and postural awareness and retraining. Phase IV included improving the hand activities in activities of daily living; maintenance of the regained function by involvement in leisure and sport activities and home programme was prescribed.

Visual Analog Scale (VAS) was used to assess the pain levels of study subjects before and after the rehabilitation.

Descriptive statistics like frequency, percentage values were used to analyse the population characteristics. Sample T test was used to evaluate the effectiveness of the sequenced rehabilitation protocol. Correlation coefficient was used to describe the correlation between variables.

## Results

### Demography

Among the 70 participants, 55 were male and 15 were female. The mean age was 34.18 years. Various demographic details of the participants were presented in Table 1. Most of the subjects were using Blackberry (52.85%), followed by ordinary mobile phone (18.57%), iPhone (12.85%) and other smart phones (10%). 35.67% of the users who were diagnosed to have MSD in the present study were primary level managers and 32.85% of the subjects were senior executives of major multi-national companies.

**Table 1:** Demographic characteristics of the participants (n = 70)

**Variable**	**N**	**%**
Gender		
Male	55	78.6
Female	15	21.4
Age group		
< 14 years	1	1.4
14 to 25 years	2	2.85
26 to 40 years	38	54.2
41 to 56 years	29	41.4
Hand Dominance		
Right	62	88.6
Left	5	11.4
Hours of Mobile Usage/day		
< 2 hours	7	10
2 to 3 hours	32	45.71
3 to 4 hours	21	30
> 4 hours	10	1.43
Type of Handheld device		
Blackberry	37	52.85
Other Smartphone	13	10
Ordinary phone	9	18.57
iPhone	7	12.85
Gaming device	4	5.7
Predominant type of usage		
Text messaging	56	80
E mail	44	63
Games	21	30
Social networking	21	30
Internet surfing	24	34

### Pain characteristics

**Figure 1:**
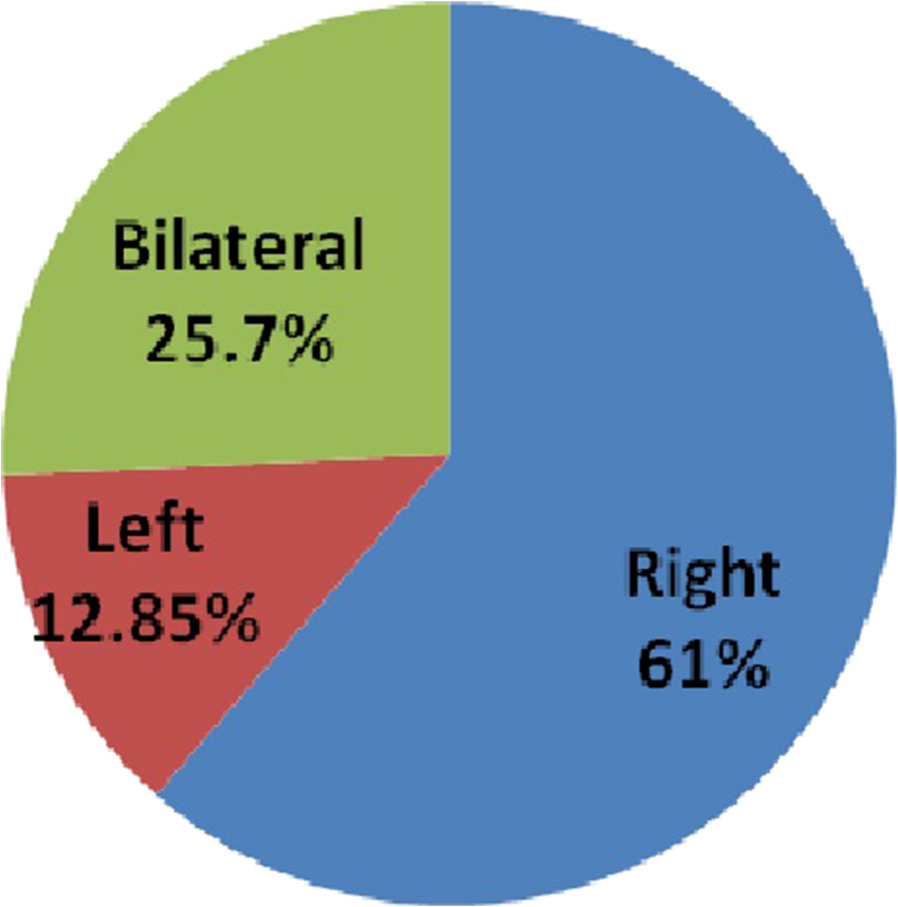
Laterality of symptoms.

Clinical assessment showed that for majority of the individuals, right side was more commonly affected (61%) when compared to the left side and bilateral involvement (Figure 1). The common symptoms reported by the subjects during examination were pain in the thumb and forearm with associated burning, numbness and tingling around the thenar aspect of the hand with stiffness of wrist and hand.

### Co-morbidities

All the subjects (n = 70) were diagnosed to have tendinosis of extensor pollicis longus and myofascial pain syndrome affecting the 1^st^ interossei, thenar group of muscles and extensor digitorum communis. The commonest associated co-morbidities were myofascial pain syndrome of neck and upper back (70.37%) and thoracic outlet syndrome (51.85%). The co-morbidities are presented in Table 2.

**Table 2:** Associated co-morbidities reported among the subjects

**Associated disorders**	**n**	**%**
Myofascial pain syndrome of neck and upper back	48	69
Thoracic outlet syndrome	34	49
Fibromyalgia syndrome	7	10
Hypothyroidism	2	2.9
Extensor wrist tendinosis	4	5.7
De Quervain’s tenosynovitis	2	2.9

### Correlation analysis

A significant positive correlation was found between the hand dominance and occurrence of upper extremity MSDs in the studied individuals using HHD (p < 0.01). After the rehabilitation following a sequenced protocol the VAS scale showed significant reduction in pain levels (p < 0.01).

## Discussion

The preliminary study by the authors on 27 subjects described the common clinical features noted in MSD of upper extremity due to HHD [12]. The finding of tendinosis of extensor pollicis longus, myofascial pain syndrome of adductor pollicis, 1^st^ interossei and extensor digitorum communis in all the subjects was replicated in the present study. Studies have revealed that while texting in mobile phone keypad, the thumb covered motions in planes of extension, flexion, abduction-adduction and opposition. These motions occurred simultaneously in three dimensions and as a result it became difficult to measure the kinematics of thumb [13]. This posture of the thumb working near the extreme range of motion was perhaps the main triggering factor for the development of tendinosis of extensor pollicis longus as reported in our study. Studies related to measurement of thumb postures during texting were shown to be affected by the size of the mobile phone and movement axes of the thumb [7],[14]. This might have been a notable factor for our study subjects who used blackberry and other smart phones which are comparatively larger and promote usage of thumb alone for texting. Static loading by holding of the hand held device for long durations, often coupled with hazardous body postures and overuse of the hand muscles are likely contributors to the development of myofascial pain syndrome of hand, forearm, neck and upper back muscles [6]. Nintendo thumb, Gamer’s grip and Nintendinitis are terms used to describe a video game related MSD, similar to the disorders occurring in text messaging, affecting the hands. The movement of fingers are similar to texting on mobile screen. Symptoms reported in earlier studies included blistering, paraesthesia and swelling of the thumbs or fingers due to tendinosis and bursitis [11].

A study showed that postures and the type of mobile phone task affected muscle activity and thumb positions [7]. The same study reported that females compared to males had higher muscle activity in the extensor digitorum communis and the abductor pollicis longus when entering SMS messages and tended to have greater thumb abduction, higher thumb movement velocities and fewer pauses in the thumb movements [7]. However, in our present study such differences were not present and most of the affected subjects were males.

The present study was a retrospective report analysis with relatively small sample size of 70 subjects. Therefore generalisation of the result is difficult and use of more detailed statistical analysis was not possible. A prospective cross sectional analysis involving a larger sample size on various types of HHD, various brands and user characteristics is recommended. This will pave way for recommending appropriate design of HHD and improving user friendliness according to the anthropometry of the users. Also, further investigation of the pathogenesis of MSDs related to usage of HHD is needed so that an effective prevention strategy can be formulated.

## Conclusions

HHD that promotes the predominant usage of thumb or only one finger while texting or gaming are associated with a higher prevalence of MSDs and hence the users are advised to select devices that are designed to permit typing or usage with all the fingers. Other preventive methods like limiting the total hours of usage of HHD, frequent short breaks between usage of HHD, maintenance of correct posture and usage of voice to text software could also be advised. The study concluded that treatment with a sequenced rehabilitation protocol was effective for MSD’s of upper extremities caused due to extensive usage of HHD. Further studies involving identification of risk factors in larger population are recommended to prevent these disorders.

### Consent

Written informed consent was obtained from the all the subjects before the evaluation for publication of this report and any accompanying images.

## Competing interests

The authors declare that they have no competing interests.

## Authors’ contributions

DS Participated in conception and design, assessment of subjects, critical review of manuscript. MM participated in the conception and design, statistical analysis, drafting and critical review of the manuscript. RR participated in data collection and critical review of the manuscript. JJ participated in data collection and critical review of the manuscript. All authors read and approved the final manuscript.
